# Establishment of Regional Concentration–Duration–Frequency Relationships of Air Pollution: A Case Study for PM_2.5_

**DOI:** 10.3390/ijerph17041419

**Published:** 2020-02-22

**Authors:** Hone-Jay Chu, Muhammad Zeeshan Ali

**Affiliations:** Department of Geomatics, National Cheng Kung University, Tainan 70101, Taiwan; zeeshanktk1992@yahoo.com

**Keywords:** PM_2.5_, duration, frequency, spatial map

## Abstract

Poor air quality usually leads to PM_2.5_ warnings and affects human health. The impact of frequency and duration of extreme air quality has received considerable attention. The extreme concentration of air pollution is related to its duration and annual frequency of occurrence known as concentration–duration–frequency (CDF) relationships. However, the CDF formulas are empirical equations representing the relationship between the maximum concentration as a dependent variable and other parameters of interest, i.e., duration and annual frequency of occurrence. As a basis for deducing the extreme CDF relationship of PM_2.5_, the function assumes that the extreme concentration is related to the duration and frequency. In addition, the spatial pattern estimation of extreme PM_2.5_ is identified. The regional CDF identifies the regional extreme concentration with a specified duration and return period. The spatial pattern of extreme air pollution over 8 h duration shows the hotspots of air quality in the central and southwestern areas. Central and southwestern Taiwan is at high risk of exposure to air pollution. Use of the regional CDF analysis is highly recommended for efficient design of air quality management and control.

## 1. Introduction

Air pollution has become a worldwide environmental health risk [1,2], especially in the developing countries. In management, an essential task is to understand the duration, frequency, and intensity of air pollution [3,4]. Poor air quality usually affects human health [5,6,7]. Elderly people, children, and people with heart or lung conditions are more sensitive than others to the effects of inhaling fine particles (PM_2.5_). Acute exposure to PM_2.5_ is associated with various health outcomes, such as respiratory symptoms [8,9], hospital admissions, or death [10]. The maximum PM_2.5_ concentrations have significant positive impacts on outpatient visits [11]. Chronic exposure to PM_2.5_ can lead to chronic diseases or reduced life expectancy [12]. The risk that extreme pollutant concentrations pose to human health is the subject of environmental concern [10].

The impact of frequency and duration of air quality has received considerable attention over the past decades. However, few people discuss the concentration–duration–frequency (CDF) relationships in air pollution. The CDF is modified from the intensity–duration–frequency (IDF) relationship, which is a mathematical relationship between the rainfall intensity, duration, and annual frequency of occurrence [13]. The most common IDF techniques from hydrological engineering were used for Gumbel distribution [14]. However, the CDF is related to the extreme concentration of air pollution with its duration and annual frequency of occurrence. The CDF formulas are empirical equations representing a relationship between the maximum concentration as a dependent variable and other parameters of interest, i.e., duration and annual frequency of occurrence. In the frequency aspect, the return period of an event might be 100 years and is expressed as its probability of occurring being 1/100, or 1% in any one year.

Many types of distributions, such as log-normal, Weibull, Pearson, gamma, and Gumbel distributions, are used to fit the distribution of air pollutant concentration [10,15,16,17,18]. The probability of occurrence and the CDF of extreme PM_2.5_ can be investigated using standard statistical techniques, e.g., Gumbel, log-normal, and Weibull probability distributions. In this study, the CDF analysis of fine aerosols was performed using the Gumbel probability distribution. The analysis system uses the actual measurement records to calculate the occurrence values of different frequencies according to the statistical properties of the samples and presumes the possible devaluation values to derive the basis of the extreme CDF relationship. Moreover, the empirical CDF relationship in this study is assumed to be the Bernard formula function [13,19]. The coefficient estimation is performed in a regression, and the estimated extreme concentration based on the defined return period and duration is finally obtained.

The CDF analysis for a single station can be derived from historical data of PM_2.5_. However, the spatial pattern of air pollution is heterogeneous [20]. The regional CDF function is developed and determined on the basis of the regional parameters. Based on interpolation, the regional parameters can be estimated. The regional CDF analysis provides spatial patterns of extreme air pollution and further provides detailed spatial patterns in areas where no observations are available. The model estimates the extreme concentrations at unknown locations by simultaneously considering the regional parameters and using the regional CDF function as proxy information to provide information on the relationships of spatial patterns of air quality with various durations and return periods. Using the regional CDF analysis, the hotspot area of air pollution with a specified duration and return period can be identified.

The objective of the study was to develop the relationship between the extreme PM_2.5_ concentration, the duration, and the frequency of occurrence. As a basis for deducing the Gumbel extreme value distribution and the empirical CDF model, the extreme concentration was assumed to be related to the duration and frequency, and the coefficient estimation was carried out in a regression. Furthermore, regional CDF estimations were conducted. It was expected that the regional CDF would identify the regional extreme concentration map with a specified duration and return period.

## 2. Materials and Study Area

**Figure 1:**
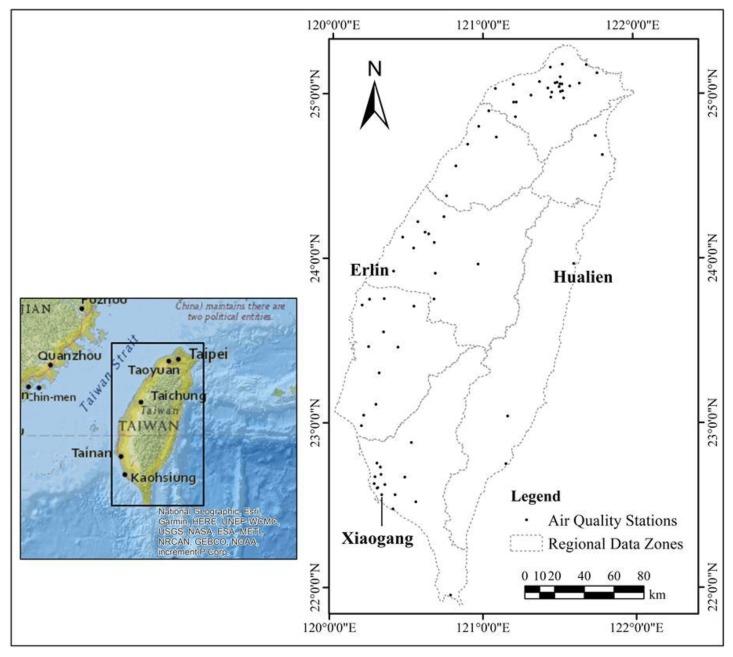
Locations of PM_2.5_ monitoring stations.

Figure 1 shows the locations of air quality monitoring stations in Taiwan. Air quality zones, e.g., northern, central, southwestern, and eastern, are also established in Taiwan (Figure 1). The Taiwanese Environmental Protection Agency (TWEPA) has been regularly recording the air quality and meteorological data throughout Taiwan since July 1982 [20]. Since August 2005, the TWEPA has completed the installation of 76 monitoring instruments for fine airborne aerosols (PM_2.5_; Figure 1). In this study, the historical data obtained by the TWEPA’s Air Monitoring Network were obtained for the PM_2.5_ hourly fine aerosol concentration in Taiwan from 2005 to 2015. This study used the hourly PM_2.5_ obtained by the TWEPA stations that are in compliance with the regulatory air monitoring procedures.

## 3. Methods

First, the moving average of PM_2.5_ data for various durations needed to be prepared. Derivations of the PM_2.5_ CDF relationships for each station for the different return periods were determined by fitting the Gumbel distribution to the corresponding maximum concentration per year at various stations (Section 3.1). The empirical model for the PM_2.5_ CDF analysis at each station was developed (Section 3.2). Eventually, the regional PM_2.5_ CDF analysis was performed (Section 3.3).

### 3.1. Derivations of PM_2.5_ CDF

The probability of occurrence of the extreme pollution event (C≥c) for the annual frequency of exceedance (F) is the inverse of the return period calculated as follows:

(1) $$P\left(C\geq c\right)=F=\frac{1}{T}$$

The probability of non-exceedance is as follows:

(2) $$P\left(C\leq c\right)=1-\frac{1}{T}$$

where T is the return period or recurrence interval of extreme concentration, C is the extreme PM_2.5_ concentration, and c is the threshold.

The Gumbel distribution was fitted to the extreme PM_2.5_ data, i.e., to the maximum annual values. The cumulative Gumbel extreme value distribution is illustrated as follows [21]:

(3) $$P\left(C\leq c\right)=e^{-e^{-\alpha \left(C-\beta \right)}}$$

where P(C≤c) is the probability of non-exceedance, and α and β are the parameters of the Gumbel distribution. From the observed sequence, the parameters can be estimated [13]. The parameters α and β are linked to the mean value ($\bar{C}$) of the extreme PM_2.5_ data and to the standard deviation ($\sigma_{C}$) of the extreme PM_2.5_ data through the following equations:

(4) $$\alpha =\frac{1.282}{\sigma_{C}}$$

(5) $$\beta =\bar{C}-\frac{0.577\sigma_{C}}{1.282}=\bar{C}-0.45\sigma_{C}$$

The logarithm was taken twice to yield a formulation from Equation (3), and Equation (2) was merged into Equation (3). Thus, the extreme concentration of PM_2.5_ (C) for each return period is as follows:

(6) $$C=\beta -\frac{1}{\alpha }\ln(\mathrm{lnT}-\ln\left(T-1\right))$$

In addition, C can be expressed as a function of the return period for each duration. The average extreme concentrations for various durations were calculated based on the aforementioned steps. For the respective periods, the maximum values of 1, 8, 16, 24, 48, and 96 h-average concentrations were used for the modeling. In the specific duration, the PM_2.5_ extreme concentration is a function of the return period. The following return periods are considered in this study: 5, 10, 20, 30, 40, 50, 60, 75, and 100 years.

### 3.2. Empirical PM_2.5_ CDF Function

Based on the above modeling, the empirical CDF formulas can be developed on the basis of the equations representing a relationship between the maximum PM_2.5_ concentration (C), duration (D), and frequency (F), which is the inverse of the return period (1/T), as follows:

(7) C=f(T,D)

where D is the duration. The empirical function follows the power expression based on the Bernard equation [13,19]:

(8) $$C=kT^{a}D^{-b}$$

where k, a, b are the model coefficients. We also consider the natural logarithm of both sides of the equation:

(9) $$lnC=lnk+alnT^{}-blnD^{}$$

The least squares method is applied to determine the parameters of the empirical CDF equation. The parameters of Equation (9) for different return periods (5, 10, 20, 30, 40, 50, 60, 75, and 100 years) and different durations (1, 8, 16, 24, 48, and 96 h) are calculated.

### 3.3. Regional CDF Mapping of Extreme PM_2.5_

**Figure A1:**
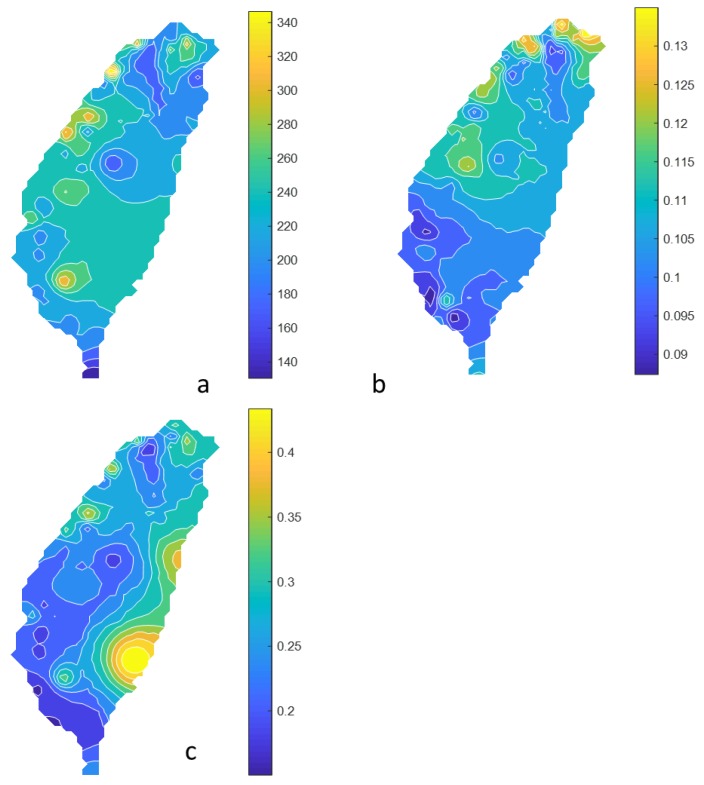
(**a**) k(x), (**b**) a(x), (**c**) b(x) coefficient maps.

The empirical PM_2.5_ CDF function for each station was identified using Equation (8), whereas the regional CDF model was determined using Equation (10). The regional PM_2.5_ CDF model is generated from the CDF with spatially varying parameters k(x), a(x), b(x) that are spatially interpolated. Regional concentration C(x) of PM_2.5_ at any location x is the function within a specified return period (T) within a duration (D) as follows:

(10) $$C\left(x\right)=k\left(x\right)T^{a\left(x\right)}D^{-b\left(x\right)}$$

(11) $$i.e.\;lnC\left(x\right)=lnk\left(x\right)+a\left(x\right)lnT^{}-b\left(x\right)lnD{,}^{}$$

where regional coefficients, e.g., k(x), a(x), b(x), at any location x are generated using k, a, b regression parameters by using the inverse distance weighted (IDW). The IDW method is a straightforward and low-computational approach for spatial interpolation procedures [22]. The k(x), a(x), b(x) regional coefficient maps are shown in the Appendix A Figure A1.

## 4. Results and Discussion

### 4.1. Descriptive Statistics and Plots of Extreme PM_2.5_ Concentration

**Figure 2:**
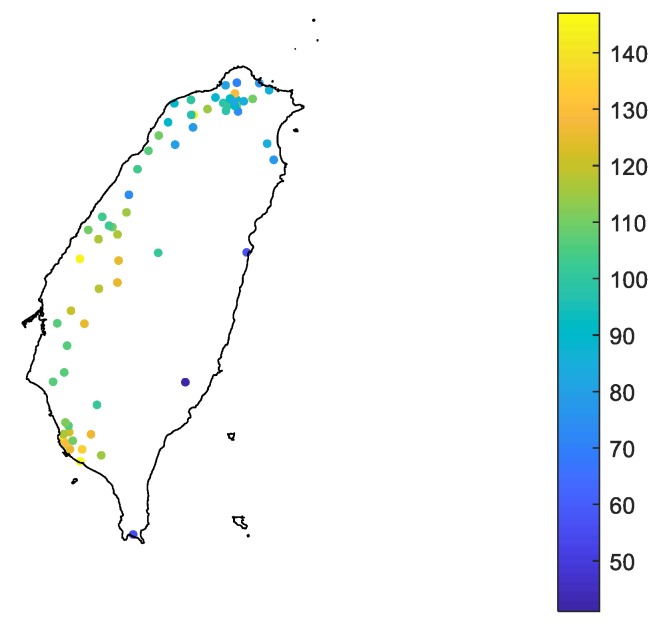
Average extreme PM_2.5_ concentration within 96 h from 2005 to 2015 (unit: μg/m^3^).

Figure 2 shows the maximum 96 h-average PM_2.5_ concentration from 2005 to 2015. The maximum, the average value, and the standard deviation of the extreme data are 147, 103, and 21 μg/m^3^, respectively. The extreme PM_2.5_ concentration is lower in northern and eastern Taiwan and higher in central and southern Taiwan. This result leads to the regionalization of the CDF relationships for various geographical areas.

**Figure 3:**
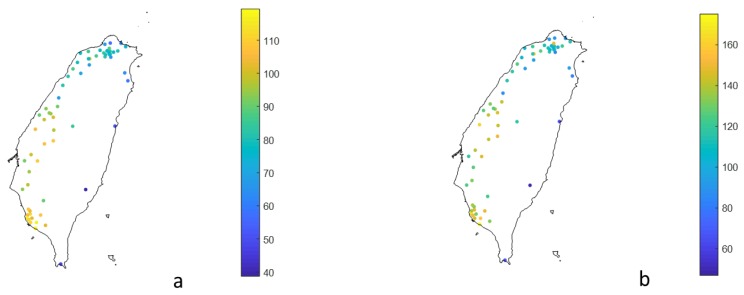
Average extreme PM_2.5_ concentration within 96 h for 5-year (**a**) and 100-year (**b**) return periods (unit: μg/m^3^).

In this study, the CDF relationship (Equations (4) to (6)) was derived for each station, and the empirical CDF formulas (Equation (8)) can be evaluated with different durations and return periods. Figure 3 shows the result plots of the extreme 96 h-average concentration at 5-year and 100-year return periods. In the comparison of various return periods, the PM_2.5_ concentration patterns are similar, but the values are different. For the 100-year return period, the maximum, the average value, and the standard deviation of the extreme 96 h-average concentrations are 175, 121, and 25 μg/m^3^, respectively. The extreme PM_2.5_ value for the 100-year return periods is 1.5 times of the 5-year ones. Pollution hotspots are in the neighboring counties and cities of central and southwestern Taiwan. Eastern Taiwan contains the lowest concentration of PM_2.5_. 

### 4.2. Heterogeneity of Air Pollution

**Figure 4:**
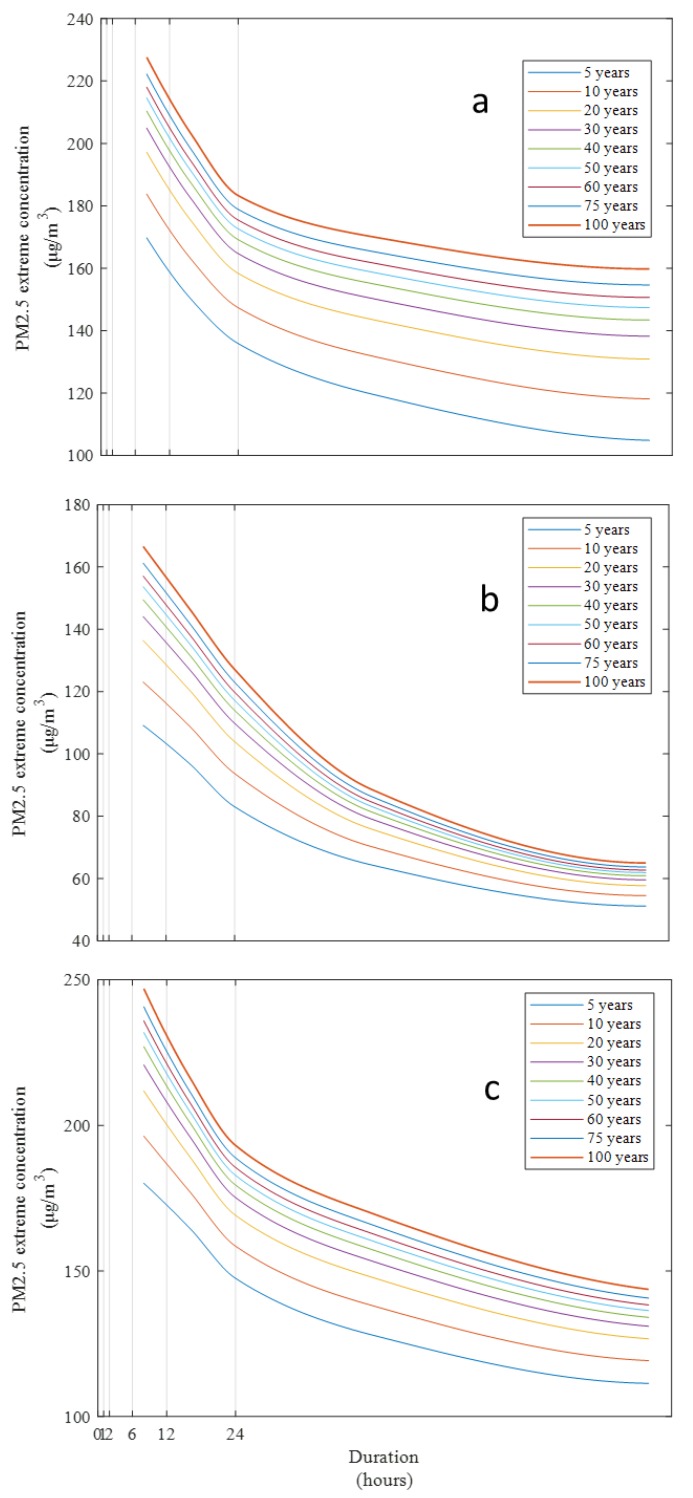
CDF curves in (**a**) Erlin, (**b**) Hualien, and (**c**) Xiaogang for the duration of 8–96 h.

According to the CDF relationship, the PM_2.5_ concentration is high with a low return period. In addition, the decrease of the extreme concentration varies with increasing duration. Figure 4 shows the comparison of the CDF curves in Erlin, Xiaogang, and Hualien in western, southwestern, and eastern Taiwan (locations shown in Figure 1). For the 5-year return period, the 8 h-average concentration is 110 μg/m^3^, and the 96 h-average concentration drops to 60 μg/m^3^ in Hualien. However, in Erlin and Xiaogang, the 8 h-average concentrations are 170 and 175 μg/m^3^, and the 96 h-average concentrations are only reduced to 100 and 110 μg/m^3^. Table 1 shows the model coefficient list and illustrates coefficient b for duration containing large differences in the observations. Duration of air pollution is higher in Erlin than in Xiaogang and Hualien. According to the data for the duration over 24 h, air pollution persists in Erlin more than in Xiaogang and Hualien. The CDF curves clearly show the differences between the extreme PM_2.5_ concentrations in the eastern and the western parts of Taiwan. In Figure 4, the downward slope of the CDF curve is greater in Hualien than in Erlin and Xiaogang. Air pollution was generally more serious in the western part when compared with the eastern part of Taiwan. The heterogeneity of extreme pollutant concentrations represents a different level of risk to people’s health [23]. The emissions were from local sources, such as traffic and industrial or agricultural activities, and from outside Taiwan [24,25]. A highly developed industry and a large population density distinguish the western plain of Taiwan. Air quality is mainly influenced by local emission sources over the southwestern and central inland cities of Taiwan, but meteorological conditions also affect air pollution dispersion in Taiwan [26]. Air quality in the urban and industrial regions of southwestern and west central Taiwan is poor in the winter [20]. However, traffic and industrial activities are low in eastern Taiwan. The air quality in eastern Taiwan is generally good. In addition, emission sources, meteorological conditions, and atmospheric boundary layers affect transmission and diffusion of air pollution [27,28]. Using the CDF curve and results of the regional CDF analysis, the effect of the PM_2.5_ emission due to the diffusion differences in various locations can be determined.

### 4.3. Regional CDF Analysis

**Figure 5:**
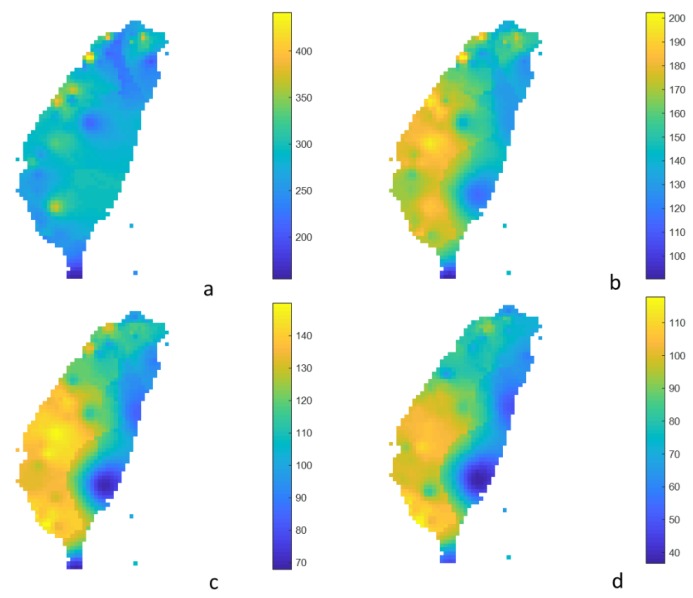
Estimated 1 h- (**a**), 8 h- (**b**), 24 h- (**c**), and 96 h- (**d**) average extreme PM_2.5_ concentration for the 5-year return period (unit: μg/m^3^).

**Figure 6:**
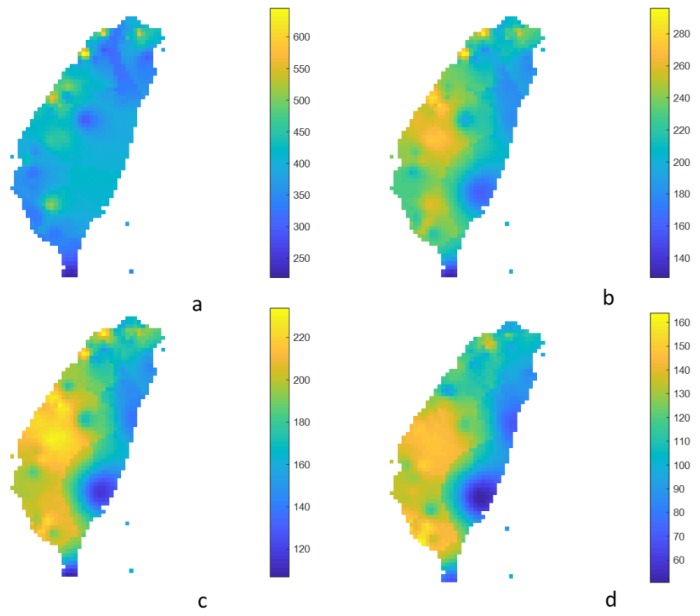
Estimated 1 h- (**a**), 8 h- (**b**), 24 h- (**c**), and 96 h- (**d**) average extreme PM_2.5_ concentration for the 100-year return period (unit: μg/m^3^).

Short- and long-return period air pollutions, such as 5-year and 100-year ones, provided the difference in frequencies of local high pollution levels and the effects of spatial PM_2.5_ patterns. Figure 5 and Figure 6 show the estimated extreme concentration for 5-year and 100-year return periods with the duration of 1, 8, 24, and 96 h. If the analyzed duration was 1 h, the few, local, and distributed hotspots were from the northern, central, and southwestern areas of Taiwan, respectively. This anomaly in air quality may have been caused by a local event, e.g., a fireworks festival, agricultural and domestic solid fuel burning, or a forest wildfire. Over 8 h, the spatial pattern of air pollution was highly similar. The greater the duration, the larger the hotspot. The PM_2.5_ exposure is not randomly distributed. The hotspots of PM_2.5_ air pollution were shown in the central area and in the southwestern area of Taiwan, especially at the 96 h duration.

The long-duration air pollution in Taiwan is usually the effect of local sources, topography, and the monsoon [20]. The current northeast monsoon is weakening, leading to poor diffusion conditions to drive air pollutants [29]. Air quality becomes poor due to stagnant conditions in western Taiwan, particularly in central and southern Taiwan. Previous research also found that elevated total ambient PM_2.5_, due to poor diffusion conditions as the northeast monsoon weakens, may be associated with acute health outcomes [30]. In general, the PM_2.5_ concentration in the summer is low, but high in winter. Air pollution in the short term (acute) and in the long term (chronic) should be considered [31,32]. Studies suggest that public health impacts of air pollution will be dominated by short-term and long-term exposure as determined by the association between exposure and mortality [6,31,33,34]. According to the spatial results, central and southwestern Taiwan is at high risk of long-duration exposure of air pollution. These areas are unhealthy for groups who are sensitive to air pollution.

Extreme pollution events were observed frequently by monitoring the network. The 1 h-average PM_2.5_ concentrations reach very high levels. Furthermore, industry and traffic emissions [35], agricultural burning [36], massive incense burning [37], dust storms [38], domestic solid fuel burning [39], and wildfires [30] significantly affect local extreme air pollution. Pollutants released into the environment are spatially fluctuating rather than uniformly distributed, and thus the health risk due to air pollution is an issue of spatial variability [23,40]. This study can provide the information on air pollution distribution with a specified return period and duration. 

### 4.4. Discussion

The CDF, or the regional CDF method, belongs to the spatiotemporal data analysis of extreme PM_2.5_ concentration data. Spatiotemporal data analysis and visualization are important for air pollution management [41]. However, the summary of the extreme pollution data, or data analysis, is critical. The CDF analysis can help us summarize the main characteristics and patterns of air pollution for a specified duration and frequency and provide information about the occurrence of limit exceedances of air pollution [10]. The CDF curves and their spatial maps can be used for area delineation, policy control, and management [42,43]. The framework is relatively practical, but the experiment proves that it is valid to analyze the extreme air pollution monitoring results with various durations and return periods. The method is used to determine the multiple functions of the CDF in the air pollution data. However, the history of records can help define the long-term behavior. We will maintain the data size as large as possible, and a future study will update the results.

Heterogeneity of PM_2.5_ is highly related to pollutant sources, geographical location, and climatic and topographic conditions [44]. The use of the regional model rather than those for individual stations logically provides more spatial information for management purposes. The extreme PM_2.5_ concentration was generally spatially varied. Moreover, the regional PM_2.5_ CDF map could be useful in order to detect the spatial variation of health risk, and the CDF of various durations is needed to determine the health risk [45]. Avoiding exposure to air pollutants is especially critical for the elderly, children, and susceptible individuals with cardiovascular or pulmonary diseases (CVD or COPD) [32]. A further study can be conducted to determine the long-duration concentrations of PM_2.5_, such as the 1-week-, 10-day-, and 1-month-average concentrations. We will apply the CDF to high-frequency and massive low-cost sensor data, such as airbox data [46,47], and identify the differences.

## 5. Conclusions

This study presented the derivation of the CDF curves of fine aerosols, PM_2.5_. The Bernard formula was applied as the basis for estimating the empirical CDF relationship. The empirical CDF curve coefficients were estimated by regressions. Moreover, the regional map for the CDF analysis can be identified based on regional parameters. Results of the preliminary analysis of the data for 2005–2015 showed that the spatial pattern of extreme air pollution was highly similar over an 8 h duration. The local pattern of air pollution lasting for 1 h may be caused by a local event, e.g., a fireworks festival, agricultural and domestic solid fuel burning, and forest wildfires. The greater the duration, the larger the hotspot. Moreover, the use of regional maps rather than those for individual stations provide spatial information on air pollution, particularly for longer durations. Using the regional extreme concentration with a specified duration and return period, the spatial pattern of air pollution showed the hotspots of air pollution in the central area and in the southwestern area of Taiwan. Central and southwestern Taiwan is at high risk of long-duration exposure of air pollution.

In addition, the CDF relationship between the stations in western and eastern Taiwan revealed air quality differences between these areas. With the same return period, the concentration was lower in the eastern than in the western part. Air pollution persisted in the western part rather than in the eastern part. Furthermore, the local and the regional CDF relationships proposed in this study can be used as a reference for health planning and management. Further study can be conducted for air quality design and control of pollution.

## Figures and Tables

**Table 1:** R^2^, root-mean-square error (RMSE), and coefficients (k, a, b) of the concentration–duration–frequency (CDF) empirical model.

	Erlin	Hualien	Xiaogang
k	182.151	283.163	259.355
a	0.118	0.108	0.092
b	0.154	0.424	0.219
R^2^	0.98	0.98	0.99
RMSE (μg/m^3^)	2.9	3.9	1.4

## Appendix A

The k(x), a(x), b(x) coefficients from Equation (10) are estimated using the IDW.
